# A mathematical model for the simulation of the contraction of burns

**DOI:** 10.1007/s00285-016-1075-4

**Published:** 2016-11-08

**Authors:** Daniël C. Koppenol, Fred J. Vermolen, Gabriela V. Koppenol-Gonzalez, Frank B. Niessen, Paul P. M. van Zuijlen, Kees Vuik

**Affiliations:** 1 Delft Institute of Applied Mathematics, Delft University of Technology, Delft, The Netherlands; 2 Department of Psychology, Education and Child Studies, Erasmus University Rotterdam, Rotterdam, The Netherlands; 3 Department of Plastic, Reconstructive and Hand Surgery, MOVE Research Institute, VU University Medical Centre, Amsterdam, The Netherlands; 4 Burn Centre, Red Cross Hospital, Beverwijk, The Netherlands; 5 Department of Plastic, Reconstructive and Hand Surgery, Red Cross Hospital, Beverwijk, The Netherlands

**Keywords:** Burns, Wound contraction, Heterogeneous, isotropic, compressible neo-Hookean solid, Multiple linear regression analysis, Probabilistic analysis, Moving-grid finite-element method, 35L65, 35M10, 62H15, 62J05, 62P30, 65C20, 68U20, 74L15, 92C10, 92C17

## Abstract

A continuum hypothesis-based model is developed for the simulation of the contraction of burns in order to gain new insights into which elements of the healing response might have a substantial influence on this process. Tissue is modeled as a neo-Hookean solid. Furthermore, (myo)fibroblasts, collagen molecules, and a generic signaling molecule are selected as model components. An overview of the custom-made numerical algorithm is presented. Subsequently, good agreement is demonstrated with respect to variability in the evolution of the surface area of burns over time between the outcomes of computer simulations and measurements obtained in an experimental study. In the model this variability is caused by varying the values for some of its parameters simultaneously. A factorial design combined with a regression analysis are used to quantify the individual contributions of these parameter value variations to the dispersion in the surface area of healing burns. The analysis shows that almost all variability in the surface area can be explained by variability in the value for the myofibroblast apoptosis rate and, to a lesser extent, the value for the collagen molecule secretion rate. This suggests that most of the variability in the evolution of the surface area of burns over time in the experimental study might be attributed to variability in these two rates. Finally, a probabilistic analysis is used in order to investigate in more detail the effect of variability in the values for the two rates on the healing process. Results of this analysis are presented and discussed.

## Introduction

Burns are a significant global problem; they constitute the second highest incidence of trauma related deaths worldwide and every year nearly half a million citizens of the US require medical treatment as a result of acute thermal injury (Gibran et al. 2013; Goel and Shrivastava 2010). In the US the majority of the injuries are minor and do not require specialized care. However, a small portion of the burn injuries are substantial and as a consequence approximately 3400 persons pass away each year and approximately 40,000 require admission to a hospital or burn center. These numbers have decreased by about 50% over the past two decades due to effective prevention strategies and advances in therapy strategies (Keck et al. 2009). This is an impressive achievement.

Nevertheless, it is in general still very difficult to prevent the development of sequelae such as the formation of permanent shortenings of scar tissue (Hawkins and Finnerty 2012). One of the main causes for this is probably a lack of knowledge about which elements of the wound healing response are the key elements in processes like wound contraction. We think that results obtained with mathematical modeling studies can provide new insights into which of these elements are key elements and that these insights might aid in the design of new treatment plans that can reduce the incidence of sequelae.

In this study we develop a mathematical model for the simulation of the contraction of burns in order to gain new insights into which elements of the wound healing response might have a substantial influence on the contraction of such wounds. For this end we first show good agreement with respect to the variability in the evolution of the surface area of burns over time between the outcomes of computer simulations obtained in this study and measurements obtained in a previously conducted experimental study. This agreement provides us some confidence about the validity of the model. In the model the variability in the evolution of the surface area of the burns over time is brought about by varying the values for some of its parameters simultaneously. In order to quantify the individual contributions of these parameter value variations to the dispersion in the surface area of healing burns and hence gain insight into which subprocesses have the largest influence on the contraction of such wounds, we subsequently use a factorial design in combination with a multiple linear regression analysis. This analysis shows that almost all variability in the surface area can be explained by variability in the value for the myofibroblast apoptosis rate and, to a lesser extent, the value for the collagen molecule secretion rate. The outcomes of the regression analysis suggest then that most of the variability in the evolution of the surface area of the healing burns over time in the experimental study might be attributed to variability in the apoptosis rate of myofibroblasts and the secretion rate of collagen molecules. We elaborate on this further in the section where we discuss the results obtained with this study.

Before presenting the model, we present in Sect. 2 a general overview of some of the elements involved in the healing of deep burns that cover a substantial surface area. Subsequently, we present the development of the model in Sect. 3. In order to generate the computer simulations a custom-made numerical algorithm had to be developed. An overview of the algorithm is presented in Sect. 4. Thereafter we present in Sect. 5 some details about the factorial design and the regression analysis that have been applied. A probabilistic analysis is also used in this study in order to investigate in more detail the effect of variability in the values for the myofibroblast apoptosis rate and the collagen molecule secretion rate on the healing of burns. More details about this probabilistic analysis are also presented in Sect. 5. Simulation results and the results obtained with the statistical analyses are presented in Sect. 6. Finally, the model and the results are discussed in Sect. 7.

## Burn wound healing: an overview

The skin is the largest organ in the human body and is essential for both the maintenance of homeostasis and the protection against potentially damaging environmental factors such as various microorganisms and applied mechanical forces (O’Toole and Mellerio 2010). When the skin is burned, skin tissue is lost rapidly due to tissue necrosis as a consequence of protein denaturation, degradation, and coagulation (Kao and Garner 2000; Keck et al. 2009). The release of local factors such as proteases and toxic oxygen byproducts, tissue edema, and alterations in blood flow may result subsequently in further injury and cell death over time. Taken together these processes cause common local complications such as infections, loss of body heat, and increased water loss.

In addition to the local injury at the site of the burn, severe thermal injury that covers a large portion of the body might also result in acute burn shock (Robins 1990) and a subsequent prolonged period of chronic systemic inflammation, hypermetabolism, and body mass wasting (Farina et al. 2013; Porter et al. 2013). Burn shock is characterized by a collection of acute systemic responses such as fluid and protein movement from the intravascular space into the interstitial space, reduced cardiac output, and hypovolemia. The systemic responses are potentially life-threatening and the delayed responses may have a huge influence on both the quality of the outcome of the wound healing process and the time required to restore the integrity of the skin (Kao and Garner 2000; Keck et al. 2009).

Given the very important functions of the skin and the complications that might arise as a consequence of (thermal) injuries, it is obvious that these injuries need to be repaired quickly and effectively. In order to accomplish this, humans have evolved sophisticated processes for the restoration of the integrity of skin. If the restoration proceeds without complications, then the final outcome of these restoration processes is not normal skin tissue in the case of deep dermal injuries such as severe burns, but whitish, thin, flat scar tissue (Hawkins and Finnerty 2012). This type of tissue is known as normotrophic scar tissue (Verhaegen et al. 2009). Although burns are different from other types of wound due to for example the aforementioned generalized effects on the body, the main processes that lead to the formation of normotrophic scar tissue are the same for all types of wound and are often divided up in three sequential, partially overlapping phases: inflammation, proliferation, and maturation/remodeling (Singer and Clark 1999; Tiwari 2012).

After an initial vasoconstriction of the blood vessels close to the site of injury, this vasoconstriction is reversed quickly and is succeeded by vasodilatation and an increased permeability of the walls of these vessels (Baum and Arpey 2005). Due to these alterations several types of leukocyte such as neutrophils, macrophages, and lymphocytes are able to actively infiltrate the site of injury. This active infiltration marks the beginning of the inflammatory phase (Lawrence 1998; Singer and Clark 1999). The leukocytes clean the wounded area by removing microorganisms and debris through phagocytosis and the release of different types of metalloproteinase (MMP) (Eming et al. 2007). Furthermore, these cells secrete a mixture of signaling molecules (i.e., growth factors and cytokines), which subsequently modulate many of the other processes of the wound healing response (Li et al. 2007).

Soon after the initiation of the inflammatory phase, the proliferative phase of the wound healing cascade starts. The main processes that take place during the proliferative phase are reepithelialization, angiogenesis, fibroplasia, and wound contraction (Baum and Arpey 2005; Singer and Clark 1999). During reepithelialization the presence of an intact epidermis at the site of injury is restored and during angiogenesis a sequence of subprocesses is executed through which the presence of blood vessels in the wounded area is restored. The former process closes the gap while the latter restores a crucial network through which nutrients, cells, oxygen, and other products can be delivered efficiently at the site of injury.

Fibroplasia encompasses the subprocesses that bring about both the restoration of a collagen-rich extracellular matrix (ECM) and the presence of a fibroblast population at the site of injury. Restoration of the presence of a fibroblast population can be accomplished through cell division of fibroblasts present in the injured area, and cell migration of fibroblasts present in the surrounding uninjured tissue into the wounded area. At the site of injury the fibroblasts can differentiate into myofibroblasts (Majno 1979; Tomasek et al. 2002). Taken together this heterogeneous population of (myo)fibroblasts is the main producer of the collagens that constitute the backbone of the newly generated ECM.

Wound contraction is the process that causes the circumferential inward movement of surrounding undamaged skin tissue towards the wounded area (O’Toole and Mellerio 2010). Due to this the surface area of the burn is reduced without the production of new material. Given that wound contraction accounts for up to 40% decrease in the size of the wounded area in the case of deep dermal injuries, wound contraction is an important component of the wound healing response (Li et al. 2007). Myofibroblasts present in the wounded area are the predominant mediators of this contraction process (Tomasek et al. 2002). Due to the presence of actin microfilament bundles within the myofibroblasts, these cells are capable of pulling on the ECM with relatively much force. Given that they are located within the injured area, this pulling at the ECM results in the inward movement of the undamaged skin tissue towards the wounded area.

With the onset of the restoration of a collagen-rich ECM the remodeling of this evolving ECM also starts (Enoch and Leaper 2007). During remodeling the nature of the ECM changes due to a gradual decrease in the cell densities of various cell types, a change in the balances between the production and the breakdown of various constituents of the ECM, and an adjustment in the way that the constituents of the ECM are aligned and interconnected (Monaco and Lawrence 2003). Taken together these alterations result in the formation of normotrophic scar tissue of gradually increasing strength (Hawkins and Finnerty 2012).

## Development of the mathematical model

Given that contraction mainly takes place in the dermal layer of the skin, solely a portion of this layer is incorporated explicitly into the model. The layer is modeled as a continuum. The neighboring dermal structures are incorporated implicitly into the model through mechanical interactions between these structures and the modeled portion of the dermal layer at the individual interfaces.

In order to allow for large strains we model the dermal layer as a heterogeneous, isotropic, compressible neo-Hookean solid (Treloar 1948). With respect to the mechanical component of the model the displacement of the dermal layer ($\mathbf {u}$) is selected as the primary model variable. In addition, we select the following four constituents of the dermal layer as primary model components: a fibroblast population (*N*), a myofibroblast population (*M*), a generic signaling molecule (*c*), and collagen molecules ($\rho$).

We use the general continuum hypothesis-based modeling framework of Tranquillo and Murray (1992) as mathematical basis for the model. This framework consists of the following general set of conservation equations:

1a $$\begin{aligned} \frac{\partial z_{i}}{\partial t} + \nabla \cdot \left[ z_{i}\mathbf {v}\right]&= -\nabla \cdot \mathbf {J}_{i} + R_{i}, \end{aligned}$$

1b $$\begin{aligned} -\nabla \cdot \varvec{\sigma }&= \mathbf {f}. \end{aligned}$$

Equation (1a) is the conservation equation for the cell density/concentration of constituent *i* of the dermal layer and Equation (1b) is the reduced conservation equation for the linear momentum of the dermal layer. Like others, we assume that the inertial forces that work on the dermal layer are negligible (Olsen et al. 1995; Tranquillo and Murray 1992). As a consequence the conservation equation for the linear momentum of the dermal layer reduces to the above force balance equation. Within the above equations, $z_{i}$ represents the cell density / concentration of constituent *i*, $\mathbf {v}$ represents the displacement velocity of the dermal layer, $\mathbf {J}_{i}$ represents the flux associated with constituent *i* per unit area, $R_{i}$ represents the chemical kinetics associated with constituent *i*, $\varvec{\sigma }$ represents the Cauchy stress tensor associated with the dermal layer, and $\mathbf {f}$ represents the total body force working on the dermal layer. Given the chosen primary model variables, we have $i \in \{N,M,c,\rho \}$. In the remainder of this text, we replace $z_{i}$ by *i*. Hence, $z_{N}$ becomes *N*, $z_{M}$ becomes *M*, and so on.

### The cell populations

The random movement of both fibroblasts and myofibroblasts through the dermal layer and the directed movement of both fibroblasts and myofibroblasts up the gradient of the signaling molecule are incorporated into the model. The random movement of the cells is modeled by means of cell density-dependent Fickian diffusion and the directed movement of the cells is modeled by means of a simple model for chemotaxis (Hillen and Painter 2009):

2 $$\begin{aligned} \mathbf {J}_{N}&= -D_{F}F\nabla N + \chi _{F}N\nabla c, \end{aligned}$$

3 $$\begin{aligned} \mathbf {J}_{M}&= -D_{F}F\nabla M + \chi _{F}M\nabla c, \end{aligned}$$

where

4 $$\begin{aligned} F = N + M. \end{aligned}$$

The parameter $D_{F}$ is the cell density-dependent (myo)fibroblast random motility coefficient and $\chi _{F}$ is the chemotactic parameter that depends on both the binding rate and the unbinding rate of the signaling molecule with its receptor and the concentration of this receptor on the cell surface of the (myo)fibroblasts. Certain members of the family of platelet derived growth factors (PDGF) are good examples of molecules that act as a strong attracting stimuli for (myo)fibroblasts (Barrientos et al. 2008).

Furthermore, we incorporate into the model the cell division of fibroblasts and myofibroblasts by using two nearly identical adjusted logistic growth models. The difference between the two growth models is that myofibroblasts solely divide in the presence of the signaling molecule while fibroblasts also divide without the presence of the signaling molecule. The actual rate of cell division of both fibroblasts and myofibroblasts is dependent on the concentration of the signaling molecule. Additionally, we incorporate into the model the cell differentiation of fibroblasts into myofibroblasts under the influence of the signaling molecule. Examples of signaling molecules that can stimulate both the up-regulation of the cell division rate and the cell differentiation rate of fibroblasts into myofibroblasts are certain members of the family of transforming growth factors $\beta$ (TGF-$\beta$) (Werner and Grose 2003). Finally, we incorporate into the model the removal of (myo)fibroblasts from the dermal layer by means of apoptosis. Taken together we obtain

5 $$\begin{aligned} R_{N}&= r_{F}\left[ 1 + \frac{r_{F}^{\max }c}{a_{c}^{I} + c}\right] [1 - \kappa _{F}F]N^{1 + q} - k_{F}cN - \delta _{N}N, \end{aligned}$$

6 $$\begin{aligned} R_{M}&= r_{F}\left[ \frac{\left[ 1 + r_{F}^{\max }\right] c}{a_{c}^{I} + c}\right] [1 - \kappa _{F}F]M^{1 + q} + k_{F}cN - \delta _{M}M, \end{aligned}$$

where $r_{F}$ is the cell division rate, $r_{F}^{\max }$ is the maximum factor with which the cell division rate can be enhanced due to the presence of the signaling molecule, $a_{c}^{I}$ is the concentration of the signaling molecule that causes the half-maximum enhancement of the cell division rate, $\kappa _{F}F$ represents the reduction in the cell division rate due to crowding, *q* is a fixed constant, $k_{F}$ is the signaling molecule-dependent cell differentiation rate of fibroblasts into myofibroblasts, $\delta _{N}$ is the apoptosis rate of fibroblasts, and $\delta _{M}$ is the apoptosis rate of myofibroblasts.

### The generic signaling molecule

We assume that both fibroblasts and myofibroblasts release and consume the signaling molecules. The functional form for these processes is based on chemical interactions between the signaling molecules and receptors for these molecules on the cell surfaces of the (myo)fibroblasts. The derivation of the functional form (i.e., the first term on the right hand side of Eq. (8)) can be found in the appendix of the article by Olsen et al. (1995). Furthermore, we incorporate into the model the removal of signaling molecules from the dermal layer through proteolytic breakdown. Finally, we assume that the signaling molecules diffuse through the dermal layer according to linear Fickian diffusion. Taken together this results in

7 $$\begin{aligned} \mathbf {J}_{c}&= -D_{c}\nabla c, \end{aligned}$$

8 $$\begin{aligned} R_{c}&= k_{c}\left[ \frac{c}{a_{c}^{II} + c}\right] \left[ N + \eta M\right] - \delta _{c}g(F,c,\rho )c, \end{aligned}$$

where $D_{c}$ is the Fickian diffusion coefficient of the generic signaling molecule, $k_{c}$ is the maximum net secretion rate of the signaling molecule, $\eta$ is the ratio of myofibroblasts to fibroblasts in the maximum net secretion rate of the signaling molecule and the collagen molecules (see the next subsection), $a_{c}^{II}$ is the concentration of the signaling molecule that causes the half-maximum net secretion rate of the signaling molecule, and $\delta _{c}$ is the proteolytic breakdown rate of the signaling molecules.

The last term of $R_{c}$ requires some more explanation. We incorporate into the model the proteolytic breakdown of the signaling molecules by a generic MMP (Mast and Schultz 1996; Van Lint and Libert 2007). It is known that MMPs are involved in the breakdown of collagen-rich fibrils during the remodeling of the ECM and the maintenance of the ECM (Chakraborti et al. 2003; Lindner et al. 2012; Nagase et al. 2006). Furthermore, it is known that (myo)fibroblasts are important producers of MMPs (Lindner et al. 2012) and that the production of MMPs can be reduced in the presence of signaling molecules like TGF-$\beta$ (Overall et al. 1991). Therefore, we assume that the concentration of the generic MMP is a function of the concentration of the collagen molecules, the concentration of the signaling molecule, and the cell density of the (myo)fibroblast population. In this study we take the following relationship:

9 $$\begin{aligned} g(F,c,\rho ) = \frac{F\rho }{1 + a_{c}^{III}c}, \end{aligned}$$

where $1/[1 + a_{c}^{III}c]$ represents the inhibition of the synthesis of the generic MMP due to the presence of the signaling molecule.

### The collagen molecules

We assume that secreted collagen molecules are attached to the ECM instantaneously. Hence we assume that no active transport of collagen molecules takes place. Furthermore, we incorporate into the model the production of collagen molecules by both fibroblasts and myofibroblasts. The secretion rate of the molecules is enhanced in the presence of the signaling molecule. Examples of signaling molecules that can upregulate the secretion of collagen molecules by (myo)fibroblasts are certain members of the family of transforming growth factors $\beta$ (TGF-$\beta$) (Werner and Grose 2003). Finally, we incorporate into the model the proteolytic breakdown of the collagen molecules analogously to the breakdown of the signaling molecules. Taken together we obtain

10 $$\begin{aligned} \mathbf {J}_{\rho }&= \mathbf {0}, \end{aligned}$$

11 $$\begin{aligned} R_{\rho }&= k_{\rho }\left[ 1 + \left[ \frac{k_{\rho }^{\max }c}{a_{c}^{IV} + c}\right] \right] \left[ N + \eta M\right] - \delta _{\rho }g(F,c,\rho )\rho , \end{aligned}$$

where $k_{\rho }$ is the collagen molecule secretion rate, $k_{\rho }^{\max }$ is the maximum factor with which the secretion rate can be enhanced due to the presence of the signaling molecule, $a_{c_{N}}^{IV}$ is the concentration of the signaling molecule that causes the half-maximum enhancement of the secretion rate, and $\delta _{\rho }$ is the degradation rate of the collagen molecules.

### The force balance

We take the following constitutive stress-strain relation:

12 $$\begin{aligned} \varvec{\sigma }&= 2C_{1}\left[ \sqrt{\text {det}(\mathbf {B})}\right] ^{-\frac{5}{3}}\left[ \mathbf {B} - \frac{1}{3}\text {tr}\left( \mathbf {B}\right) \mathbf {I}\right] + 2D_{1}\left[ \sqrt{\text {det}(\mathbf {B})} - 1\right] \mathbf {I}, \end{aligned}$$

13 $$\begin{aligned} \mathbf {B}&= \left[ \mathbf {I} - \nabla \mathbf {u} - \left[ \nabla \mathbf {u}\right] ^{\text {T}} + \left[ \nabla \mathbf {u}\right] ^{\text {T}}\nabla \mathbf {u}\right] ^{-1}, \end{aligned}$$

14 $$\begin{aligned} C_{1}&= \frac{E\rho }{4[1 + \nu ]}, \end{aligned}$$

15 $$\begin{aligned} D_{1}&= \frac{E\rho }{6[1-2\nu ]}, \end{aligned}$$

where $\mathbf {B}$ is the left Cauchy–Green deformation tensor, $\mathbf {I}$ is the second order identity tensor, and $\nu$ is Poisson’s ratio (Treloar 1948). Like Ramtani (2004) and Ramtani et al. (2002), we assume that the Young’s modulus of the tissues is dependent on the concentration of the collagen molecules. In this study we assume that this dependence is linear. Hence we take $E\rho$ for the Young’s modulus, where *E* is a constant.

Furthermore, we incorporate into the model the forces that are generated by the myofibroblasts due to their pulling on the ECM (Hinz 2007). In this study we model the pulling force as an isotropic stress that is proportional to the product of the cell density of the myofibroblast population and a simple function of the concentration of the collagen molecules (Olsen et al. 1995). No other forces are incorporated into the model. Taken together we obtain

16 $$\begin{aligned} \mathbf {f} = \nabla \cdot \left[ \xi M\left[ \frac{\rho }{R_{\rho }^2 + \rho ^{2}}\right] \mathbf {I}\right] , \end{aligned}$$

where $\xi$ is the generated stress per unit cell density and the inverse of the unit collagen molecule concentration, and $R_{\rho }$ is a fixed constant.

### The domain of computation

**Fig. 1:**
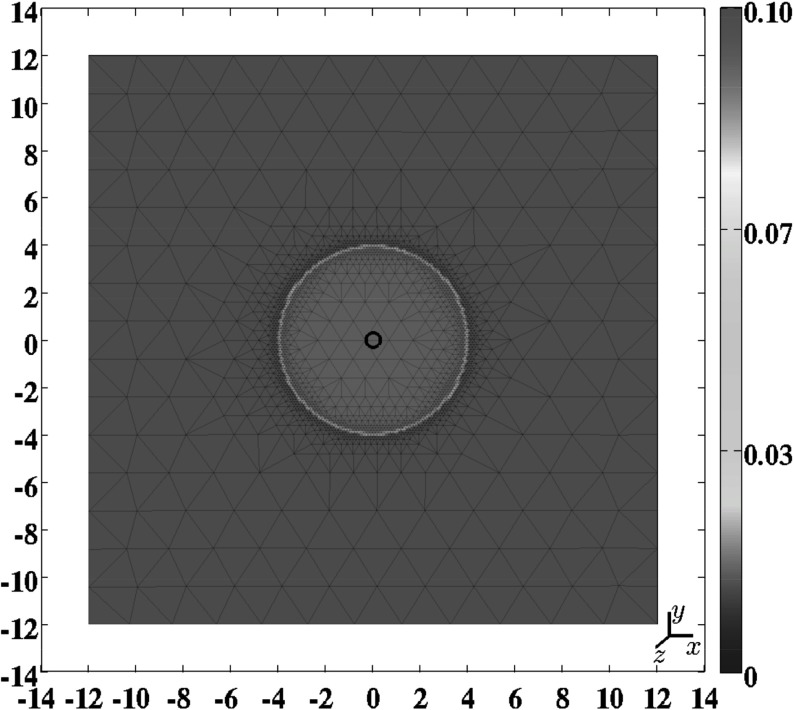
A graphical representation of the domain of computation (the *scale* along both axes is in centimeters). Depicted are the initial shape of the modeled portion of dermal layer and, in color scale, the initial concentration of the collagen molecules (with $I^{w} = 10^{-1}$), measured in $\text {g}/\text {cm}^{3}$. The black circle located more or less at the center of the wound, marks the material point within the dermal layer where the evolution of the concentrations (cell densities) of the different modeled constituents was traced over time for the generation of some of the figures in Sect. 6

We assume that $\partial u /\partial z = \partial v /\partial z = w = 0$ holds within the modeled portion of the dermal layer, with the *xy*-plane running parallel to the surface of the skin and $\mathbf {u} = (u,v,w)^{\text {T}}$. Effectively this implies that the plane strain assumption holds within the portion of dermal layer (Lai et al. 1999). Furthermore, we assume that the derivatives of the concentrations and the cell densities of the modeled constituents are equal to zero in the direction perpendicular to the surface of the skin. Hence $\partial i /\partial z = 0$ with $i \in \{N,M,c,\rho \}$. Taken together these assumptions imply that the calculations for obtaining simulations can be performed on an arbitrary, infinitely thin slice of dermal layer oriented parallel to the surface of the skin and that the results from these calculations are valid for every infinitely thin slice of dermal layer oriented parallel to the surface of the skin. For the generation of simulation results we used the computational domain depicted in Fig. 1. Using Lagrangian coordinates ($\mathbf {X} = (X,Y,Z)^{\text {T}}$), the domain of computation (${\varOmega }_{X}$) is described mathematically by

17 $$\begin{aligned} {\varOmega }_{X} \in \{-12 \le X \le 12,\ -12 \le Y \le 12,\ Z = 0\}. \end{aligned}$$

### The initial conditions and the boundary conditions

The initial conditions give a description of the different cell densities and concentrations at the onset of the proliferative phase of the wound healing cascade. For the generation of simulation results the following general indicator function has been used to describe the shape of the burn:

18 $$\begin{aligned} w\left( \mathbf {X},c^{I},c^{II}\right) = {\left\{ \begin{array}{ll} 0 &{} \text {if } \left\| \mathbf {X}\right\| < \left[ c^{I} - c^{II}\right] , \\ \frac{1}{2}\left[ 1 + \sin \left( \frac{\left[ \left\| \mathbf {X}\right\| - c^{I}\right] \pi }{2c^{II}}\right) \right] &{} \text {if } \left| \left\| \mathbf {X}\right\| - c^{I}\right| \le c^{II}, \\ 1 &{} \text {if } \left\| \mathbf {X}\right\| > \left[ c^{I} + c^{II}\right] . \end{array}\right. } \end{aligned}$$

Here $w = 0$ corresponds to completely wounded dermis and $w = 1$ corresponds to unwounded dermis. The values for the parameters $c^{I}$ and $c^{II}$ determine respectively the radius of the initial burn and the steepness of the boundary of the wounded area (i.e., the minimum distance between completely wounded dermis and unwounded dermis). We have chosen for a circular shape so that the shape of the burns in the computer simulations is equal to the shape of the burns that were created in the experimental study by Wang et al. (2011). Certain measurements obtained in this experimental study were used for comparison against certain outcomes of the obtained computer simulations in this study (see Sect. 6).

**Fig. 2:**
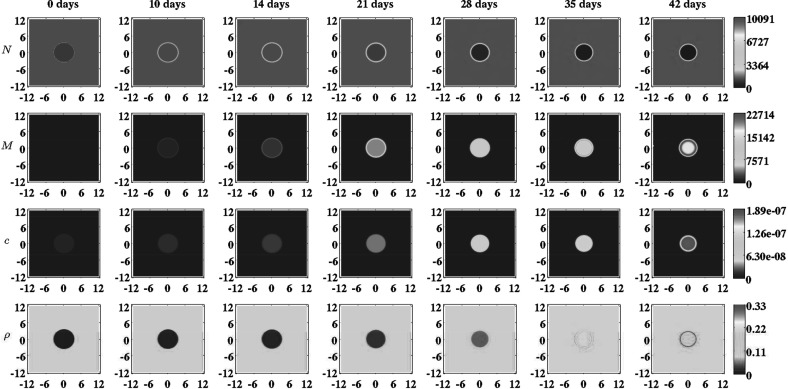
An overview of a simulation with a relatively small burn ($c^{I} = 3.57\ \text {cm}$), a relatively low minimum initial cell density of fibroblasts and a relatively low minimum initial concentration of collagen molecules ($I^{w} = 1\times 10^{-1}$), a relatively low apoptosis rate of myofibroblasts ($\delta _{M} = 2\times 10^{-2}\ /\text {day}$), and an average collagen molecule secretion rate ($k_{\rho } = 1.75\times 10^{-8}\ \text {g}/(\text {cells day})$). The *first two rows* show the evolution over time of the cell density of respectively the fibroblast population and the myofibroblast population. The *color scales* represent the cell densities, measured in $\text {cells}/\text {cm}^{3}$. The *last two rows* show the evolution over time of the concentration of respectively the signaling molecules and the collagen molecules. The *color scales* represent the concentrations, measured in $\text {g}/\text {cm}^{3}$. Within the subfigures, the scale along both axes is in centimeters. The values for the remaining parameters are equal to those depicted in Table 1

Based on the general function for the shape of the wound we take the following initial conditions for the modeled constituents of the dermal layer:

19 $$\begin{aligned} N(\mathbf {X},0)&= \left[ I^{w} + \left[ 1 - I^{w}\right] w\left( \mathbf {X},c^{I},c^{II}\right) \right] \overline{N}, \nonumber \\ M(\mathbf {X},0)&= \overline{M}, \nonumber \\ c(\mathbf {X},0)&= \left[ 1 - w\left( \mathbf {X},c^{I},c^{II}\right) \right] c^{w}, \nonumber \\ \rho (\mathbf {X},0)&= \left[ I^{w} + \left[ 1 - I^{w}\right] w\left( \mathbf {X},c^{I},c^{II}\right) \right] \overline{\rho }, \end{aligned}$$

where $\overline{N}$, $\overline{M}$, and $\overline{\rho }$ are respectively the equilibrium cell density of the fibroblast population, the equilibrium cell density of the myofibroblast population, and the equilibrium concentration of the collagen molecules, of the unwounded dermis. See Fig. 1 for a detailed graphical representation of the initial concentration of the collagen molecules and see the leftmost column of Fig. 2 for graphical representations of all initial cell densities and concentrations. Due to early secretion of signaling molecules by for instance leukocytes, signaling molecules are present at the site of injury. The constant $c^{w}$ is the maximum of the initial concentration of the signaling molecule in the wounded area. Furthermore, we assume that there are some fibroblasts and collagen molecules present in the wounded area; the parameter $I^{w}$ determines the minimum amount of fibroblasts and collagen molecules that are present initially in the wounded area. Finally, we take $3.57 \le c^{I} \le 3.99\ \text {cm}$ and $c^{II} = 0.10\ \text {cm}$.

With respect to the initial conditions for the displacement of the dermal layer, the following holds. The initial cell density of the myofibroblast population is equal to zero everywhere in the domain of computation. Looking at Eq. (16), this implies $\mathbf {f}(\mathbf {x},0) = \mathbf {0}$. Hence

20 $$\begin{aligned} \mathbf {u}(\mathbf {x},0) = \mathbf {0}\quad \forall \mathbf {x} \in {\varOmega }_{x}, \end{aligned}$$

where ${\varOmega }_{x}$ is the domain of computation in Eulerian coordinates and $\mathbf {x} = (x,y,z)^{\text {T}}$ are the Eulerian coordinates.

With respect to the boundary conditions for the modeled constituents of the dermal layer, we take the following Dirichlet boundary conditions

21 $$\begin{aligned} N = \overline{N},\ \ M = \overline{M},\ \ c = \overline{c}, \end{aligned}$$

where $\overline{c}$ is the equilibrium concentration of the signaling molecules in the unwounded dermis. With respect to the boundary conditions for the mechanical component of the model, we take the following Robin boundary condition

22 $$\begin{aligned} \mathbf {n}\cdot \varvec{\sigma } = -s\rho \mathbf {u}, \end{aligned}$$

where $\mathbf {n}$ is the unit outward pointing normal vector to the boundary of the domain of computation. This boundary condition implies that the boundaries experience a spring-like force per unit area in the opposite direction of the displacement of the dermal layer that is proportional to the concentration of the collagen molecules and the magnitude of this displacement.

**Table 1:** An overview of the dimensional (ranges of) values for the parameters of the model

Parameter	Value	Dimensions	References
$D_{F}$	$$10^{-7}\ $$	$\text {cm}^{5}/(\text {cells day})$	Sillman et al. (2003)
$\chi _{F}$	$$2\times 10^{-3}\ $$	$\text {cm}^{5}/(\text {g day})$	Murphy et al. (2012)
*q*	$$-4.2\times 10^{-1}\ $$	−	NC
$r_{F}$	$$9.24\times 10^{-1}\ $$	$\text {cm}^{3q}/(\text {cells}^{q}\ \text {day})$	Ghosh et al. (2007)
$r_{F}^{\max }$	$$2\ $$	−	Strutz et al. (2001)
$a_{c}^{I}$	$$10^{-8}\ $$	$\text {g}/\text {cm}^{3}$	Grotendorst (1992)
$\kappa _{F}$	$$10^{-6}\ $$	$\text {cm}^{3}/\text {cells}$	Van de Berg et al. (1989)
$k_{F}$	$$5.4\times 10^{6}\ $$	$\text {cm}^{3}/(\text {g day})$	Desmoulière et al. (1993)
$\delta _{N}$	$$2\times 10^{-2}\ $$	$/\text {day}$	Olsen et al. (1995)
$\delta _{M}$	$$(1 - 6)\times 10^{-2}\ $$	$/\text {day}$	TW
$D_{c}$	$$2.9\times 10^{-3}\ $$	$\text {cm}^{2}/\text {day}$	Murphy et al. (2012)
$k_{c}$	$$4\times 10^{-13}\ $$	$\text {g}/(\text {cells day})$	Olsen et al. (1995)
$\eta$	$$2\ $$	−	Rudolph and Vande Berg (1991) & Moulin et al. (1998)
$a_{c}^{II}$	$$10^{-8}\ $$	$\text {g}/\text {cm}^{3}$	Olsen et al. (1995)
$\delta _{c}$	$$5\times 10^{-4}\ $$	$\text {cm}^{6}/(\text {cells g day})$	Olsen et al. (1995)
$a_{c}^{III}$	$$2\times 10^{8}\ $$	$\text {cm}^{3}/\text {g}$	Overall et al. (1991)
$k_{\rho }$	$$(1.25 - 2.75)\times 10^{-8}\ $$	$\text {g}/(\text {cells day})$	TW
$k_{\rho }^{\max }$	$$10\ $$	−	Olsen et al. (1995)
$a_{c}^{IV}$	$$10^{-9}\ $$	$\text {g}/\text {cm}^{3}$	Roberts et al. (1986)
$\delta _{\rho }$	$$(1.25 - 2.75)\times 10^{-6}\ $$	$\text {cm}^{6}/(\text {cells g day})$	NC
*E*	$$1.00\times 10^{2}\ $$	$(\text {N cm})/\text {g}$	Liang and Boppart (2010)
$\nu$	$$4.9\times 10^{-1}\ $$	−	Liang and Boppart (2010)
$\xi$	$$5\times 10^{-3}\ $$	$(\text {N g})/(\text {cells cm}^{2})$	Maskarinec et al. (2009) & Wrobel et al. (2002)
$R_{\rho }$	$$3\times 10^{-1}\ $$	$\text {g}/\text {cm}^{3}$	Olsen et al. (1995)
$\overline{N}$	$$10^{4}\ $$	$\text {cells}/\text {cm}^{3}$	Olsen et al. (1995)
$\overline{M}$	$$0\ $$	$\text {cells}/\text {cm}^{3}$	Olsen et al. (1995)
$\overline{c}$	$$0\ $$	$\text {g}/\text {cm}^{3}$	NC
$\overline{\rho }$	$$10^{-1}\ $$	$\text {g}/\text {cm}^{3}$	Olsen et al. (1995)
$I^{w}$	$$(1 - 2)\times 10^{-1}\ $$	−	TW
$c^{w}$	$$10^{-8}\ $$	$\text {g}/\text {cm}^{3}$	Olsen et al. (1995)
*s*	$$10^{3}\ $$	$\text {N}/\text {g}$	Koppenol et al. (2016)

The last column contains the references to the articles that were used for obtaining (estimates of) the values for the parameters. If the range of the value for a parameter was estimated in this study, then this is indicated by the abbreviation TW. If the value for a parameter is a necessary consequence of the values chosen for the other parameters, then this is indicated by the abbreviation NC

### The (ranges of the) values for the parameters

Table 1 provides an overview of the dimensional (ranges of the) values for the parameters of the model. The majority of these values were either obtained directly from previously conducted studies or estimated from results of previously conducted studies. In addition, we were able to determine the values for three more parameter due to the fact that these values are a necessary consequence of the values chosen for other parameters. These are the equilibrium signaling molecule concentration of the unwounded dermis ($\overline{c}$), the constant *q*, and the collagen molecule degradation rate ($\delta _{\rho }$). If we take $\overline{\rho } = 0.1 \ \text {g}/\text {cm}^{3}$, $\overline{N} = 10^{4}\ \text {cells}/\text {cm}^{3}$, and $\overline{M} = 0\ \text {cells}/\text {cm}^{3}$ in the unwounded dermis, then $c = 0\ \text {g}/\text {cm}^{3}$ is an attracting equilibrium in the vicinity of $c = 0\ \text {g}/\text {cm}^{3}$. Hence we take $\overline{c} = 0\ \text {g}/\text {cm}^{3}$. Furthermore, if $\overline{N} = 10^{4}\ \text {cells}/\text {cm}^{3}$, $\overline{M} = 0\ \text {cells}/\text {cm}^{3}$, and $\overline{c} = 0\ \text {g}/\text {cm}^{3}$, then $q = [\log (\delta _{N}) - \log (r_{F}[1 - \kappa _{F}\overline{N}])]/\log (\overline{N})$. Finally, if $\overline{M} = 0\ \text {cells}/\text {cm}^{3}$ and $\overline{c} = 0\ \text {g}/\text {cm}^{3}$, then $\delta _{\rho } = k_{\rho }/\overline{\rho }^2\ \text {cm}^{6}/(\text {cells g day})$.

For completeness, we want to remark here that there is (some) variability in the values for most of the parameters that were either estimated from results of previously conducted studies or obtained directly from previously conducted studies. However, given the complexity of the model and hence the necessary complexity of the custom-made numerical algorithm, we decided to set the values for these parameters to fixed values within the found ranges during the generation of simulation results in order to prevent the total computation time of the statistical analyses from rising too high.

The ranges of the values for the free parameters $\delta _{M}$, $k_{\rho }$, and $I^{w}$ were chosen in such a way that there is good agreement with respect to the variability in the evolution of the surface area of burns over time between the outcomes of computer simulations obtained in this study and measurements obtained in a previously conducted experimental study by Wang et al. (2011) (see Sect. 6). Finally, the range of the value for the radius of the initial burn (i.e., the range of the value for the parameter $c^{I}$ (see Sect. 3.6)) was chosen in such a way that it matches with the range of the radii of the burns that were created during the experimental study of Wang et al. (2011).

### A qualitative description of the dynamics of the model

Here we give a qualitative description of how the healing of a burn is accomplished in the presented model and how the different subprocesses that have been incorporated into the model, interact with each other. Due to the presence of signaling molecules in the wounded area at the onset of the proliferative phase fibroblasts from the surrounding uninjured tissue are attracted towards the wounded area. Within the wounded area the fibroblasts proliferate and due to the presence of the signaling molecules, the rate of proliferation is enhanced. In addition, the signaling molecules stimulate the cell differentiation of fibroblasts into myofibroblasts. This results in the emergence of a myofibroblast population in the wounded area. While there are signaling molecules present, both fibroblasts and myofibroblasts also secrete signaling molecules which results subsequently in a further enhancement of both the cell differentiation rate and the cell proliferation rate of fibroblasts and myofibroblasts and hence a further growth of the size of the myofibroblast population within the wounded area. The present (myo)fibroblasts in the wounded area produce collagen molecules in order to restore the presence of a collagen-rich ECM and due to the presence of the signaling molecules the rate of production of the collagen molecules is enhanced. Furthermore, the present myofibroblasts in the wounded area pull on the ECM and as a consequence the surface area of the burn is reduced slowly over time.

During the execution of the wound healing processes the concentration of the generic MMP rises slowly in the recovering wounded area and due to this the secretion of signaling molecules becomes smaller than the proteolytic breakdown of these molecules at a certain point in time. This results in a relatively fast disappearance of the signaling molecules from the wounded area and as a consequence of that the rate of production of collagen molecules starts to decline, as does the rate of cell proliferation and cell differentiation. Slowly the cell densities and the concentrations in the recovering wound area will approach the equilibrium cell densities and the equilibrium concentrations of the unwounded area due to, respectively, cell apoptosis and proteolytic breakdown of the molecules, and ultimately the properties of the recovering wounded area will become identical to the properties of the surrounding tissue, which subsequently implies that the size of the surface area of the recovering wound area slowly returns to the surface area of the burn at the onset of the proliferative phase. The decline of the cell density of the myofibroblast population in the dermal layer, the decline of the concentrations of both the signaling molecules and the collagen molecules, the gradual rise of the cell density of the fibroblast population in the wounded area, and the gradual retraction of the recovering wounded area are not (clearly) visible in the results presented in Sect. 6, but these phenomena are properties of the model and are clearly visible in computer simulations where longer healing times are simulated.

## Numerical algorithm

In this section we present an overview of the custom-made numerical algorithm that had to be developed for the generation of computer simulations. The development of this algorithm was necessary in order to guarantee the positivity of the approximations of the solutions for the constituents of the dermal layer, “catch” the local dynamics of the model, and obtain sufficiently accurate simulations sufficiently fast.

For the kernel of the concrete expression of the algorithm we used MATLAB together with MATLAB’s Parallel Computing Toolbox (The MathWorks Inc 2014). Furthermore, we used a slightly adapted version of the mesh generator developed by Persson and Strang (2004), the element resolution refinement / recoarsement tool of the computational fluid dynamics (CFD) software package FEATFLOW2 (Turek 1998), and the scaling and permutation routine HSL_MC64 (HSL 2013) by interfacing these products with the kernel. Finally, we applied the following non-dimensionalisation to the model:

23 $$\begin{aligned} x&= Lx^{*}, \quad t = \left[ L^{2}/\left[ D_{F}\overline{N}\right] \right] t^{*}, \quad \rho = \overline{\rho }\rho ^{*}, \nonumber \\ N&= \overline{N}N^{*}, \quad M = \overline{N}M^{*}, \quad c = c^{w}c^{*}, \nonumber \\ \mathbf {u}&= L\mathbf {u}^{*}, \quad \mathbf {v} = \left[ \left[ D_{F}\overline{N}\right] /L\right] \mathbf {v}^{*}, \quad \varvec{\sigma } = \left[ \left[ \xi \overline{N}\right] /\overline{\rho }\right] \varvec{\sigma }^{*}, \end{aligned}$$

where $L = 1\ \text {cm}$ is the length scale of the model. The variables with the asterisks are the non-dimensionalised variables.

In the following two subsections we present a step-by-step description of the algorithm. Basically the algorithm consists of two parts. The first part of the algorithm is dedicated to the generation of a proper triangulation of the domain of computation and is described in Sect. 4.1. The second part of the algorithm is described in Sect. 4.2 and is dedicated to obtaining an approximation of the solution for the displacement and the modeled constituents of the dermal layer from Eq. (1) after application of the non-dimensionalisation.

### Generation of the initial triangulation of the domain of computation

The first part of the algorithm consists again of two subparts. First, a conforming base triangulation is generated and subsequently the element resolution refinement / recoarsement tool is used to adjust the resolution of the elements of this base triangulation.

In order to create a conforming base triangulation the aforementioned slightly adapted version of the mesh generator developed by Persson and Strang (2004) is used. This results in very high quality triangulations of the domain of computation that consist mainly of equilateral triangles. Most of the triangles that are not equilateral, are located near the left and right boundary of the domain of computation. These latter triangles are nearly equilateral. Using the following measure for the quality of a triangle *ABC*:

24 $$\begin{aligned} \alpha (ABC) = 2\sqrt{3}\left[ \frac{\Vert CA \times CB\Vert }{\Vert CA\Vert ^{2} + \Vert AB\Vert ^{2} + \Vert BC\Vert ^{2}}\right] , \end{aligned}$$

we observed that $\alpha > 0.86$ for all triangles in the generated triangulations that were used for the generation of the simulation results, where $0 \le \alpha \le 1$ (Lo 1989).

**Fig. 3:**
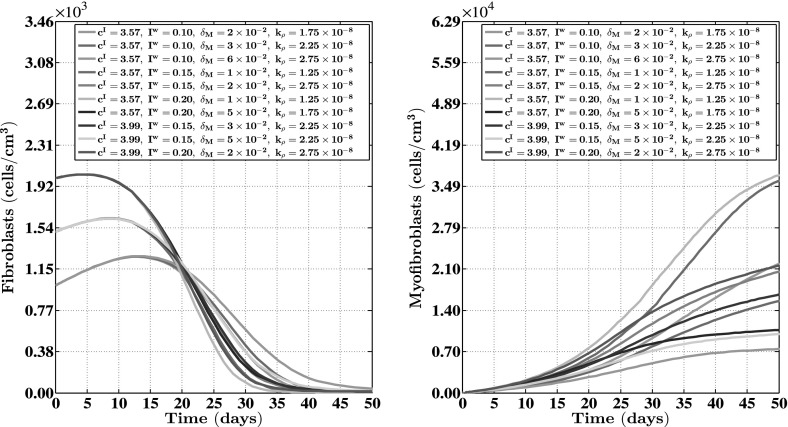
The evolution over time of the cell densities for a random selection of some combinations of the levels of the factors of the factorial design. See Fig. 1 for the location where the evolution of the cell densities was traced over time. The values for the remaining parameters are equal to those depicted in Table 1

**Fig. 4:**
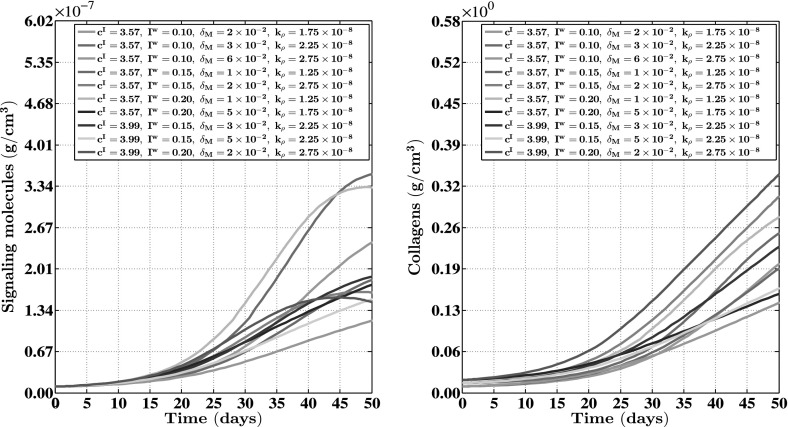
The evolution over time of the concentrations of the signaling molecules and the collagen molecules for a random selection of some combinations of the levels of the factors of the factorial design. See Fig. 1 for the location where the evolution of the concentrations was traced over time. The values for the remaining parameters are equal to those depicted in Table 1

**Fig. 5:**
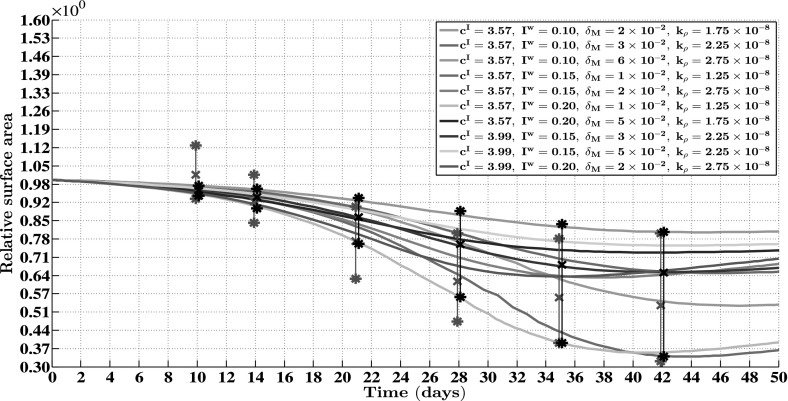
The evolution over time of the relative surface area of burns for a random selection of some combinations of the levels of the factors of the factorial design. The values for the remaining parameters are equal to those depicted in Table 1. The surface area of a burn was compared to its surface area at day 0 at every time point. The *black bars* represent the ranges of the relative surface areas of all burns at different time points after injury. The *asterisks* represent the extreme values of all combinations of the levels of the factors and the crosses represent the mean values of all combinations of the levels of the factors. Furthermore, the *red bars* represent experimental measurements of the relative surface areas of healing burns at different time points after injury in a porcine burn model (Wang et al. 2011). The *asterisks* and the *crosses* represent, respectively, the extreme values and the mean values of these measurements

**Fig. 6:**
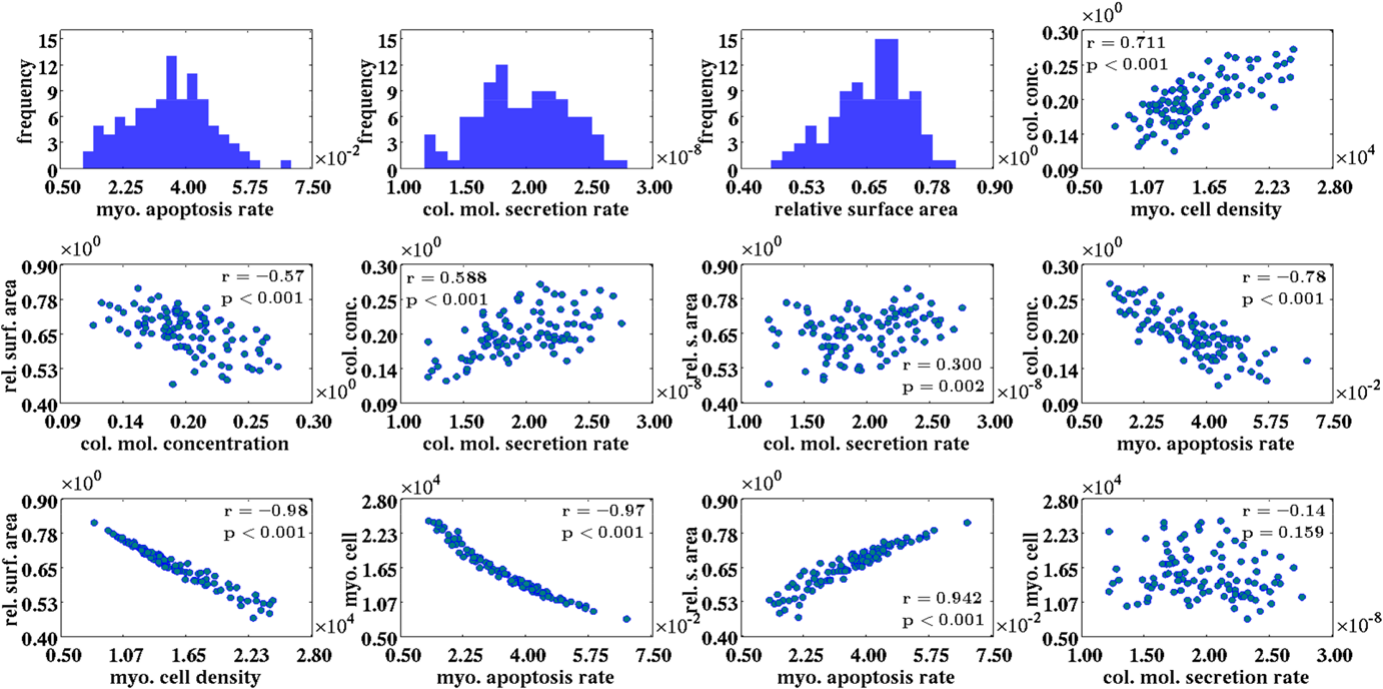
An overview of the results obtained with the probabilistic analysis. The frequency histograms display the drawn samples for the myofibroblast apoptosis rate ($\delta _{M}$) and the collagen molecule secretion rate ($k_{\rho }$), and the obtained distribution of the relative surface area of the healing burns at day 42 compared to their surface areas at day 0. The *scatter plots* provide furthermore graphical representations of the relationships between various properties of the healing burn at day 42. Within the individual subfigures the Pearson correlation coefficients are displayed together with the associated *p* values

For the generation of the simulation results presented in Figs. 2, 3, 4, 5 and 6, we used a base triangulation that consists of triangles with an average initial edge length of $1.85\ \text {cm}$. We repeated the calculations for the generation of the simulation results presented in Figs. 2, 3, 4 and 5 two times. The first time we used a base triangulation that consists of triangles with an average initial edge length of $9.24 \times 10^{-1}\ \text {cm}$ and the second time we used a base triangulation that consists of triangles with an average initial edge length of $3.46\ \text {cm}$. We observed that the difference in the simulation results with respect to the outcomes between the different calculations were small.

After generation of the conforming base triangulation the element resolution refinement / recoarsement tool is used to adjust the resolution of the elements of this base triangulation (Möller 2008). First, the $L_{2}$-norm of an estimation of the error of the gradient of the numerical approximation of the indicator function presented in Eq. (18), per element (i.e., $\left\| \hat{\mathbf {e}}\right\| _{L_{2}(T)}$) is determined (Möller and Kuzmin 2006). Thereafter, the resolution of the elements is adapted in order to adjust the estimated error. These two steps are repeated until either the absolute value of the relative change of the sum of the $L_{2}$-norm of the estimation of the error of the gradient over all elements is smaller than 5%, or the maximum number of allowed for iterations is reached. In this study we set this latter number to ten. The elements are refined and coarsened until the following holds for each element in the triangulation:

25 $$\begin{aligned} 0.04 \le \left[ \frac{\left| \mathscr {T}_{h}\right| \left\| \hat{\mathbf {e}}\right\| _{L_{2}(T)}^{2}}{{\sum \limits _{T \in \mathscr {T}_{h}}}\left( \left\| \sigma _{h}\right\| _{L_{2}(T)}^{2} + \left\| \hat{\mathbf {e}}\right\| _{L_{2}(T)}^{2}\right) }\right] ^{\frac{1}{2}} \le 0.2, \end{aligned}$$

where $\mathscr {T}_{h}$ represents the current triangulation, $\left| \mathscr {T}_{h}\right|$ denotes the number of elements that constitute the triangulation, and $\left\| \sigma _{h}\right\| _{L_{2}(T)}$ denotes the $L_{2}$-norm of a low-order estimation of the gradient of the numerical approximation of the indicator function presented in Eq. (18) (Möller and Kuzmin 2006). In this study the resolution of every individual element can be increased at most four times and the size of the individual elements cannot be increased beyond the size they have in the base triangulation.

### Determination of the approximation of the solution

As was mentioned before, the second part of the algorithm is dedicated to obtaining an approximation of the solution for the displacement and the modeled constituents of the dermal layer from Eq. (1). In order to solve the time-dependent problem the method of lines and the standard fixed-point defect correction method are applied (Van Kan et al. 2014). The two equations of the system are solved in a segregated way. Each time step approximations of the solutions for the modeled constituents of the dermal layer are determined first and subsequently an approximation of the solution for the displacement of the dermal layer is determined. This scheme is iterated until the maximum of the relative 1-norms of the residuals of the approximations is smaller than one, and the maximum of the relative 1-norms of the difference between subsequent approximations per variable is smaller than $5 \times 10^{-2}$. If the fixed-point scheme does not meet the convergence criteria within five iterations, then the scheme is interrupted, the time step is decreased to 85% of its current value, and subsequently the scheme is restarted. The required estimate of the gradient of the solution for the signaling molecule is obtained by using a variational gradient recovery projection scheme (Lyra 1994).

For the discretization of the system of equations, a moving-grid finite-element method is used (Madzvamuse et al. 2003) together with the first-order backward Euler time-integration method. Furthermore, a semi-implicit flux-corrected transport (FCT) limiter developed by Möller et al. (2008), and a source term splitting procedure proposed by Patankar (1980), are applied on the discretized system of equations that describe the dynamics of the modeled constituents of the dermal layer. Taken together these latter two techniques enforce positivity of the approximations of the solutions for the constituents of the dermal layer.

The individual time steps are chosen by using an automatically adaptive time-stepping method with inbuilt local truncation error control (Kavetski et al. 2002). The maximum size of the initial time step is set to $10^{-5}$ dimensionless units and the upper bound of the size of the time step is set to $10^{-3}$ dimensionless units. If a time step is accepted, then the subsequent time step is at most 1.25 times the size of the current time step. If a time step is rejected, then the subsequent time step is at least 0.25 times the size of the current time step. For completeness we mention here also that we set the absolute and relative truncation error tolerance to, respectively, $10^{-2}$ and $5 \times 10^{-2}$ dimensionless units (see the article by Kavetski et al. (2001) for further details on this matter). After obtaining and accepting an approximation for a certain time step, the local extrapolation procedure proposed by Kavetski et al. (2002) is applied to increase the accuracy of the approximation.

Furthermore, the aforementioned element resolution refinement / recoarsement tool is applied every ten time steps to adjust the resolution of the elements of the triangulation. For this end the $L_{2}$-norm of an estimation of the error of the gradient of the numerical approximation of the solution for the concentration of the collagen molecules per element is determined first (Möller and Kuzmin 2006). Thereafter the resolution of the elements is adapted in a fashion identical to the procedure described in Sect. 4.1 in order to adjust the estimated error. For the interpolation of approximations to new nodes in the triangulation, piecewise bivariate Hermite interpolation is used (Feng and Zhang 2013). The required gradients of the approximations at the existing nodes are estimated by using a polynomial preserving gradient recovery scheme (Zhang and Naga 2005).

For the approximation of the individual primary variables of the model functions from the space of triangular finite-elements with linear basis functions were chosen (Quarteroni and Valli 2008). The integrals over the interior of the elements are approximated by a second-order accurate Newton-Cotes quadrature rule and the integrals over the boundaries of the elements are approximated by a second-order accurate Gaussian quadrature rule.

In order to obtain approximate solutions for the resulting linear systems of equations, MATLAB’s backslash operator is used (The MathWorks Inc 2014) after using the LU factorization algorithm (Davis and Duff 1997) on scaled and permuted versions of the original linear systems. For the scaling and permutation of the linear systems several inbuilt scaling and permutation algorithms of MATLAB are used (Davis et al. 2004; Duff and Koster 1999) together with the scaling and permutation routine HSL_MC64 (HSL 2013).

## Details of the applied statistical methods

### The factorial design and the regression analysis

In this study we used a simple mixed-level full-factorial design in combination with a multiple linear regression analysis in order to quantify the individual contributions of the variations in the values for the free parameters of the model and the initial radius of the wound, to the dispersion in the surface area of healing burns (Phadke 1989; Tabachnick and Fidell 2007; Taguchi 1987). We chose the relative surface area of the healing burn at day 42 compared to the surface area of the burn at day 0 as the response variable (i.e., the dependent variable). The free parameters of the model and the initial radius of the wound were chosen as factors (i.e., independent variables). These latter parameters were assigned discrete values (i.e., levels) that divide the range of each factor equally. Here we assigned two levels to the radius of the initial burn ($c^{I}$), three levels to the minimum amount of fibroblasts and collagen molecules that are present initially in the wounded area ($I^{w}$), four levels to the collagen molecule secretion rate ($k_{\rho }$), and six levels to the myofibroblast apoptosis rate ($\delta _{M}$). For the analysis all possible combinations of the levels of the factors were examined. Hence in total 144 computer simulations were generated.

The obtained simulation data were analyzed in IBM SPSS by means of the regression analysis (IBM Corp 2011). No interactions or powers of independent variables were included in this analysis. In order to reduce the skewness and kurtosis of the data related to the relative surface area of the healing burn and to improve the normality, linearity, and homoscedasticity of the residuals of the regression analysis, the Rankit rank-based normalization method was used on the data related to the response variable. Furthermore, all data related to the factors of the factorial design were transformed to standard scores (i.e., *z* values).

The surface area of a the burns was determined in two steps. In the first step we determined the displacements of the material points with coordinates $(c^{I}(\cos (2\pi (i-1)/40)),c^{I}(\sin (2\pi (i-1)/40)),0)^{\text {T}}$, where $i \in \{1,\ldots ,40\}$. Subsequently, the area of the polygon with vertices located at the displaced material points was computed. This area is approximately equal to the surface area of the healing burn.

### The probabilistic analysis

After having used a regression analysis, a probabilistic analysis was used in order to investigate in more detail the effect of variability in the values for the collagen molecule secretion rate and the myofibroblast apoptosis rate, on both the cell density of the myofibroblasts and the concentration of the collagen molecules at day 42, and the relative surface area of the healing burn at day 42 compared to its surface area at day 0. The collagen molecule secretion rate and the myofibroblast apoptosis rate were chosen because the multiple linear regression analysis demonstrated that varying the value for these two factors has by far the largest impact on the relative surface area of the healing burn (See the presentation of the results of the linear regression analysis in Table 2).

The distribution of the collagen molecule secretion rate ($k_{\rho }$) was defined as a Gaussian (normal) distribution with a mean value of $2 \times 10^{-8}\ \text {g}/(\text {cells day})$ and a standard deviation of $3.75 \times 10^{-9}\ \text {g}/(\text {cells day})$. The distribution of the myofibroblast apoptosis rate ($\delta _{M}$) was defined as a Gaussian (normal) distribution with a mean value of $3.50 \times 10^{-2}\ /\text {day}$ and a standard deviation of $1.25 \times 10^{-2}\ /\text {day}$. The values for the parameters related to the minimum amount of fibroblasts and collagen molecules that are present initially in the wounded area ($I^{w}$) and the radius of the initial burn ($c^{I}$) were set to, respectively $1.5 \times 10^{-1}$ and $3.78\ \text {cm}$. The values for all remaining parameters are equal to those depicted in Table 1.

**Table 2:** An overview of the results obtained with the multiple linear regression analysis.

Variable	$\beta$	$t\ \text {score}$	$p\ \text {value}$	95% conf. interval		$sr^{2}$
				Lower	Upper	
$c^{I}$	0.048	2.258	0.026	0.006	0.090	0.002
$I^{w}$	$-$0.073	$-$3.463	0.001	$-$0.115	$-$0.032	0.005
$\delta _{M}$	0.912	42.978	<0.001	0.869	0.953	0.832
$k_{\rho }$	0.313	14.754	<0.001	0.271	0.355	0.098

The regression coefficients ($\beta$) are the weights of the linear regression line. The *t* scores and *p* values are the associated statistics. The squared semipartial correlations between the dependent variable and the individual independent variables ($sr^{2}$) are displayed in the last column of the table

Sampling from the Gaussian distributions was realized by using MATLAB’s inbuilt function normrnd (The MathWorks Inc 2014). In this study two samples consisting of 100 observations, were drawn from each distribution. Hence in total 100 computer simulations were generated. We used the Anderson–Darling test to investigate the degree of normality of the samples (Anderson and Darling 1952). Both samples did not show a significant deviation from a normally distributed sample ($p > 0.5$ for both samples).

The collagen molecule concentration and the cell density of the myofibroblasts were determined in two steps. In the first step we determined the cell density and the concentration at the material points with coordinates $(0,0,0)^{\text {T}}$, $((c^{I}/2)(\cos (2\pi (i-1/2)/4)),(c^{I}/2)(\sin (2\pi (i-1/2)/4)),0)^{\text {T}}$, and $(c^{I}(\cos (2\pi (i-1)/4)),c^{I}(\sin (2\pi (i-1)/4)),0)^{\text {T}}$, where $i \in \{1,\ldots ,4\}$. Subsequently, the averages of these values were calculated and these averages were used for the analysis. See Sect. 5.1 for a description of how the surface area of the healing burns was determined.

## Results

In this section we present simulation results and the outcomes of the statistical analyses. The discussion about the simulation results and the results obtained with the statistical analyses takes place in Sect. 7.

In order to obtain some insight into the dynamics of the model, we present an overview of a simulation in Fig. 2. Furthermore, Figs. 3 and 4 show, respectively, the evolution over time of the different cell densities and the evolution over time of the different concentrations for a random selection of some combinations of factor levels (i.e., some combinations of values for the initial radius of the burn, the minimum initial cell density of fibroblasts and minimum initial concentration of collagen molecules, the apoptosis rate of myofibroblasts, and the collagen molecule secretion rate), at a certain material point within the healing burn. Finally, Fig. 5 shows the evolution over time of the relative surface area of the burns for the same random selection of combinations of levels as was used for the creation of Figs. 3 and 4.

Table 2 provides an overview of the outcomes of the multiple linear regression analysis. The multiple correlation (*R*) is significantly different from zero, $F(4,139) = 520,473$, $p < 0.001$, with the squared multiple correlation at 0.937. The adjusted squared multiple correlation of 0.936 indicates that more than 93% of the variability in the relative surface area of the healing burn at day 42 compared to the size of the burn at day 0 is predicted by variability in the values for the factors of the factorial design.

Figure 6 shows an overview of the results obtained with the probabilistic analysis. The obtained distribution of the relative surface area of the healing burns at day 42 compared to their surface areas at day 0 is negatively skewed and differs significantly from a Gaussian distribution according to the Anderson–Darling test ($p < 0.04$) (Anderson and Darling 1952). The mean of the relative sizes of the healing burns at day 42 is 0.657 and the standard deviation of the obtained distribution is 0.073.

## Discussion

For approximately the last 25 years the mathematical modeling of processes involved in the healing of dermal wounds has been an active area of research. Over these years the number of generated models has increased considerably. Surveys of these models such as those compiled in Sherratt and Dallon (2002), Buganza Tepole and Kuhl (2013), and Valero et al. (2015b) indicate that the majority of the models can be placed into one of three categories: continuum hypothesis-based models, discrete cell-based models, and hybrid models. We used the general continuum hypothesis-based modeling framework of Tranquillo and Murray (1992) as mathematical basis for the generation of the presented model. Since its initial presentation this framework has been extended and adapted in several modeling studies investigating the impact of the addition and the adaptation of various different components of the wound healing response (Javierre et al. 2009; Murphy et al. 2012; Olsen et al. 1995; Ramtani 2004; Ramtani et al. 2002; Valero et al. 2013, 2014a, b, 2015a; Vermolen and Javierre 2012).

The biochemical components of the model presented in this study are based primarily on the biochemical components of the model developed by Olsen et al. (1995). However, there are also some clear differences between the model presented in this study and the model developed by Olsen et al. For instance, while in the model of Olsen et al. solely the fibroblasts are actively motile, we incorporate in our model the active movement of both fibroblasts and myofibroblasts. Thampatty and Wang (2007) have demonstrated previously that both fibroblasts and myofibroblasts are actively motile. Hence it seems reasonable to incorporate this phenomenon into the model.

In addition, we model the random movement of cells by means of cell density-dependent Fickian diffusion instead of linear Fickian diffusion. All of the models in the above cited modeling studies use linear diffusion to model the random dispersal of cells. As is also mentioned by Olsen et al. (1995), this is the standard representation of random cell movement. However, Hillen and Painter (2009) point out that it is actually far more likely that the random movement of cells depends on the density of these cells. They also show in their study that this type of dependence can show up naturally during the derivation of a Keller-Segel type of system. Hence we assume that the random movement of the cells increases with an increasing cell density (Hillen and Painter 2009; Kowalczyk 2005).

Furthermore, we incorporate into the model the degradation of both signaling molecules and collagen molecules by means of proteolytic cleavage by a generic MMP instead of by means of natural decay or very general enzymatic degradation which are usually used in this type of models. For this end, we use the phenomenological relationship given in Eq. (9) which is based on the deduction presented in the accompanying paragraph.

Finally, with respect to the biochemical components of the model, we want to place some remarks concerning the way that the cell differentiation of fibroblasts into myofibroblasts has been incorporated into the presented model. Similar to Olsen et al. (1995), we assume that the rate of cell differentiation is dependent on the concentration of the signaling molecule with no cell differentiation taking place in the absence of this signaling molecule. Like others such as Javierre et al. (2009), Murphy et al. (2012), and Valero et al. (2014a; b), we are aware of the fact that this cell differentiation can only take place under conditions of sufficient mechanical stiffness. It seems like a good idea then to incorporate this phenomenon also into the model. However, as is clearly pointed out by Van de Water et al. (2013), it is unclear at present what the actual stiffness is that is perceived by fibroblasts. In addition, recent experimental studies have demonstrated that the cell differentiation is also critically dependent on the presence of particular isoforms of fibronectin (Van de Water et al. 2013). Taken together, this implies that the incorporation of the cell differentiation mechanism into a mathematical model in a realistic way is basically not possible at present. Hence while being aware of the fact that the cell differentiation process is a very complex process in reality, we had to keep things simple in this modeling study.

With respect to the mechanical component of the presented model, we want to remark the following. Traditionally, the dermis is treated as a linear (visco)elastic solid in mechano-chemical continuum models for (certain aspects of) dermal wound healing (Javierre et al. 2009; Murphy et al. 2012; Olsen et al. 1995; Ramtani 2004; Ramtani et al. 2002; Valero et al. 2014a, b; Vermolen and Javierre 2012). However, skin tissues are actually nonlinear, anisotropic, viscoelastic, and inhomogeneous materials (Fung 1993). Hence the constitutive stress-strain relationships for skin tissues are in reality far more complicated. In order to make the presented model somewhat more realistic while still keeping the model relatively simple, we decided to model the dermal layer as a heterogeneous, isotropic, and compressible neo-Hookean solid (Treloar 1948) where the Young’s modulus is linearly dependent on the concentration of the collagen molecules (Ramtani 2004; Ramtani et al. 2002). Although we are not the first to model dermal tissues as hyperelastic materials within the context of dermal wound healing (See for example the studies by Valero et al. (2013; 2015a)), it is not a standard approach. Finally, we want to mention here that, to the best of our knowledge, we are the first to combine an explicit description of the dynamics of a myofibroblast population with the modeling of a dermal tissue as a hyperelastic material.

Ultimately the properties of the recovering wound area will become identical to the properties of the surrounding tissue (i.e., the cell densities and the concentrations in the recovering wound area will approach the equilibrium cell densities and the equilibrium concentrations of the unwounded dermis). Given Eq. (16), this implies that the “body force” in the mechanical force balance will vanish when the solutions related to the constituents of the dermal layer reach their equilibrium solutions. This implies that the displacement field of the dermal layer will become zero when the solutions related to the constituents of the dermal layer reach their equilibrium solutions and therefore the properties of the recovering tissue will indeed become identical to the properties of the surrounding tissue.

Hence it is not possible with the presented model to simulate the often observed permanent deformation of involved tissues and the development of residual stresses within these tissues (Schouten et al. 2012). Recently continuum hypothesis-based models have been formulated that are based on a morphoelastic framework (Bowden et al. 2016; Murphy et al. 2011). With these models it is possible to simulate both the contraction and the growth of involved tissues during the execution of the overall healing process. Due to this combination it is possible with this type of model to simulate the permanent deformation of involved tissues and the development of residual stresses within these tissues. Currently we are working on an extension of the model presented in this study whereby we make use of a morphoelastic framework.

Focusing now on the simulation of the contraction of burns over time we observe the following. The bars in Fig. 5 show that the agreement with respect to the variability in the evolution of the surface area of burns over time between the outcomes of the computer simulations obtained in this study and measurements obtained in a previously conducted experimental study by Wang et al. (2011) is quite good. There is in general a reasonably good match between the outcomes of the computer simulations and the measurements obtained in the experimental study with respect to both the ranges of the relative surface areas of the healing burns and the means of these relative surface areas, and this match becomes better as healing progresses. Furthermore, we also observed in the computer simulations that while some healing burns continue to contract until day 50, others stopped to contract at an earlier day and started to retract. This variability is also present in the presented results by Wang et al. (2011).

Taken together these agreements provide us some confidence about the validity of the model. Obviously the number of models with which it is possible to produce the depicted contraction curves is infinite in theory. Therefore, it would have been valuable to validate the presented model against both more experimental data and against experimental data of a different type in order to increase our confidence about the validity of the model. Unfortunately we have not been able to find any other suitable experimental data for the validation of the presented model in the available literature. However, it is a fundamental problem in the field of mathematical modeling of dermal wound healing processes to find suitable experimental data for the validation of models. As is pointed out by Bowden et al. (2016), as a consequence of technical difficulties related to obtaining reliable experimental measurement data there are hardly any suitable experimental data available at present for the proper validation of developed mathematical models in this field. In our opinion this does not mean that we should refrain from deducing biomedical implications from the results obtained with the presented model. However, we do think that it is very important to be extremely careful when doing so and to keep in mind that these implications are based on results obtained with a mathematical model.

Having said that, we look more closely now at the results presented in Figs. 3, 4 and 5. Figures 3 and 4 show clearly that varying the values for the free parameters of the model and the radius of the initial burn has in general a huge impact on the evolution over time of the cell densities and concentrations of the modeled constituents of the dermal layer within the wounded area. The dispersion in the cell density of the myofibroblast population at the center of the healing burn becomes quite large over time, and this is also the case for the dispersion in the concentration of the signaling molecules and the collagen molecules. Figure 5 shows furthermore that the evolution over time of the observed dispersion in the relative surface areas of the healing burns from the experimental study by Wang et al. (2011) can be replicated quite well by the model through the variation of the values for radius of the initial burn and the free parameters of the model.

Combined these results provide us the following explanation for the observed dispersion in the relative surface areas of the healing burns in the experimental study by Wang et al. (2011). Variability in the values for the free parameters of the model results in an increasing dispersion in both the cell density of the myofibroblast population and the concentrations of the signaling molecules and collagen molecules over time. Given Eq. (16), this implies that the dispersion in the total generated stress by the myofibroblast population also increases over time, which subsequently results in an increasing dispersion in the relative surface areas of the healing burns over time. Hence the observed variability in the evolution of the surface area of the healing burns over time in the experimental study by Wang et al. (2011) might be attributed to variability in the myofibroblast apoptosis rate, the collagen molecule secretion rate, the minimum amount of fibroblasts and collagen molecules that are present initially in the wounded area, and the radius of the initial burn.

Looking more closely at the outcomes of the multiple linear regression analysis provided in Table 2, it can be observed that all regression coefficients differ significantly from zero. Hence variability in the value for each factor of the factorial design contributes significantly to variability in the relative surface area of a healing burn at day 42. The sizes of the squared semipartial correlations indicate that variability in the value for the myofibroblast apoptosis rate has a very large impact on the relative surface area of the healing burn at day 42. Variability in the value for the collagen molecule secretion rate has a smaller, but still quite substantial impact on the relative surface area of a healing burn at day 42. Variability in the value for the parameter related to the minimum amount of fibroblasts and collagen molecules that are present initially in the wounded area, and variability in the value for the radius of the initial burn have only a relative small impact on the relative surface area of the healing burn.

Taken together the regression analysis suggests that the observed variability in the evolution of the surface area of the healing burns over time in the experimental study by Wang et al. (2011) might be attributed mainly to variability in the myofibroblast apoptosis rate and the collagen molecule secretion rate and to a far lesser extent to variability in the minimum amount of fibroblasts and collagen molecules that are present initially in the wounded area and the radius of the initial burn. Hence if one wishes to adjust the degree of wound contraction during the healing of a burn, then the simulation results and the associated regression analysis presented in this study suggest that this may be accomplished by adjusting the apoptosis rate of myofibroblasts and / or the secretion rate of collagen molecules.

We expect that varying the values for most of the remaining parameters of the model that were not included into the factorial design and for which we could find a range of values in the literature, would probably also contribute significantly to variability in the relative surface area of a healing burn at day 42. We think that varying the values for the proliferation rate of (myo)fibroblasts ($r_{F}$), the generated stress per unit cell density and the inverse of the unit collagen molecule concentration ($\xi$), and the constant *E* related to the Young’s modulus within the found ranges of values in particular would contribute to variability in the evolution of the relative surface area of a healing burn over time. Therefore, we would like to add these parameters to the factorial design in order to quantify their contribution to this variability. However, as was mentioned before, given the complexity of the model and hence the necessary complexity of the custom-made numerical algorithm, we basically had to set the individual values for these parameters to fixed values within their respective ranges in order to prevent the total computation time of the statistical analyses from rising too high.

Looking at the results obtained with the probabilistic analysis in Fig. 6, we can make a couple of interesting observations. Firstly, there is a good agreement between the regression coefficients for the collagen molecule secretion rate and the myofibroblast apoptosis rate in the linear regression line on the one hand, and the calculated correlation coefficients for the relationship between the relative surface area of a burn at day 42 and the collagen molecule secretion rate and the myofibroblast apoptosis rate on the other hand. Secondly, it is interesting to observe that the obtained distribution of the relative surface areas of the healing burns differs significantly from a Gaussian (normal) distribution, even though the samples for the collagen molecule secretion rate and myofibroblast apoptosis rate were both drawn from a Gaussian distribution, and to observe that this distribution is negatively skewed. Hence relatively speaking, only a small portion of the burns contracts severely while the majority of the burns contract relatively little. This coincides nicely with clinical observations.

Finally, it is interesting to observe that all of the remaining individual correlations displayed in the separate scatter plots make sense intuitively with the exception of the scatter plot that displays the correlation between the collagen molecule secretion rate and the cell density of the myofibroblasts. For example, we would expect positive correlations between the concentration of the collagen molecules and the rate of secretion of these molecules, and between the concentration of the collagen molecules and the cell density of the myofibroblast population. However, taking some of the correlations together shows something peculiar; while the correlation between the collagen molecule secretion rate and the concentration of the collagen molecules, and the correlation between the collagen molecule secretion rate and the relative surface area of a burn at day 42 are both positive, the correlation between the concentration of the collagen molecules and the relative surface area of a burn at day 42 is negative.

In order to explain this phenomenon, we suggest the following. Besides influencing the total amount of generated stress directly through the relationship given in Eq. (16), the concentration of the collagen molecules also influences the amount of generated stress indirectly through the following chain of connections. Given Eq. (9), it is seems reasonable to expect a positive correlation between the concentration of the collagen molecules and the concentration of the generic MMP. Given Eqs. (8) and (6), it also seems reasonable to presume a negative correlation between the concentration of the generic MMP and the concentration of the signaling molecule and a positive correlation between the concentration of the signaling molecule and the cell density of the myofibroblast population. Taken together, these correlations suggest a negative correlation between the collagen molecule secretion rate and the cell density of the myofibroblast population. Although not significantly different from zero, this is exactly what is displayed in the scatter plot that displays the correlation between the collagen molecule secretion rate and the cell density of the myofibroblasts. Figure 6 displays finally a very strong negative correlation between the cell density of the myofibroblast population and the relative surface area of a burn at day 42. We suggest that this chain of connections has a stronger effect on the relative surface area of a burn at day 42 than the direct connection between the concentration of the collagen molecules and the total amount of generated stress. As a consequence the correlation between the concentration of the collagen molecules and the relative surface area of a burn at day 42 is negative while the the correlation between the collagen molecule secretion rate and the relative surface area of a burn at day 42 is positive.

As has been mentioned in the introduction, we think that results obtained with mathematical modeling studies can provide new insights into which elements of the wound healing response are key elements in processes like wound contraction, and that these insights might aid in the design of new treatment plans for burns that can reduce the incidence of sequelae. In this study we found that most of the variability in the evolution of the surface area of healing burns over time might be attributed to variability in the apoptosis rate of myofibroblasts primarily and, to a lesser extent, the secretion rate of collagen molecules. Interestingly, this result is in agreement with the suggestion put forward by Desmoulière et al. (1995) that the disruption of apoptosis (i.e., a low apoptosis rate) during wound healing might be a very important factor in the development of pathological scars such as severely contracted scars. Hence, while we keep in mind that we are deducing biomedical implications from results obtained with a mathematical model and therefore need to be extremely careful, the results from this study suggest that the degree of wound contraction during the healing of a burn can be adjusted by altering the apoptosis rate of myofibroblasts and / or the secretion rate of collagen molecules. We find this an interesting insight and hope that this insight contributes to the design of better treatment plans for burns.
